# Efficacy and safety of dexmedetomidine in patients receiving mechanical ventilation: Evidence from randomized controlled trials

**DOI:** 10.1002/prp2.658

**Published:** 2020-11-11

**Authors:** Qinghua Dong, Chunlai Li, Fei Xiao, Yubo Xie

**Affiliations:** ^1^ Department of Anesthesiology The First Affiliated Hospital of Guangxi Medical University Nanning China

**Keywords:** dexmedetomidine, mechanical ventilation, randomized controlled trials

## Abstract

At present, the efficacy and safety of dexmedetomidine in patients receiving mechanical ventilation (MV) is still controversial. Therefore, the purpose of this research was to assess the efficacy and safety of dexmedetomidine in MV patients by reviewing the results of randomized controlled trials (RCT). RCTs evaluating the efficacy of dexmedetomidine in the treatment of MV patients were obtained by searching relevant online databases, including PubMed, EMbase, Web of Science, the Cochrane Library, Medline, OVID, and ClinicalTrials.gov. Literature meeting the inclusion criteria were selected and evaluated by two researchers independently. Risk ratio (RR)/standardized mean difference (SMD) and 95% confidence interval (CI) were used to express the differences between groups. Seven RCTs were included in our study, with 986 participants in the dexmedetomidine group and 862 participants in the control group. Summary analysis results displayed no reduction in 30‐day mortality (RR = 0.77, 95% CI: 0.59 to 1.02), delirium (RR = 0.77, 95% CI: 0.57 to 1.03), and adverse events (RR = 1.06, 95% CI: 0.22 to 5.08) in the dexmedetomidine group compared with the control group. As the length of stay in the intensive care unit (ICU) were presented as median and interquartile range (IQR)/standard deviation (SD), descriptive analysis of the results were performed. Generally, for 99.65% (953/986) of patients, dexmedetomidine was not better than the control group in reducing ICU length of stay. Our results demonstrate that for patients requiring MV, dexmedetomidine was not superior to the control group. However, analysis of more RCTs is required to confirm this conclusion.

**Abbreviations**
CI: confidence interval
ICU: intensive care unit
IQR: interquartile range
MV: mechanical ventilation
RCT: randomized controlled trials
RR: risk ratio
SD: standard deviation
SMD: standardized mean difference

## 1 INTRODUCTION

For patients receiving treatment in intensive care unit (ICU), studies have shown that sedation therapy^1^, ^2^, ^3^ could reduce the subjective discomfort caused by intervention, increase the tolerance to mechanical ventilation,^4^, ^5^ and reduce incidence of accidental removal of ventilation catheters, and finally reduce metabolic requirements during cardiovascular and respiratory instability.^6^ However, long‐term sedation has been reported to lead to serious complications, such as prolonged mechanical ventilation (MV), delusional memory and cognitive impairment,^7^ and even prolonged hospitalization,^8^ increased hospital costs and mortality.^9^ Therefore, the selecting appropriate sedation strategy is of utmost importance to improve the prognosis and quality of life of patients treated in ICU.

Dexmedetomidine is a highly efficient and highly selective α_2_‐adrenergic receptor agonist,^10^, ^11^ which is widely used in clinical surgical anesthesia and intensive care unit (ICU) sedation because of its good analgesic, sedative, and anti‐sympathetic effects.^12^ Moreover the stress response and the release of stress hormones such as cortisol is lowered by the anti‐sympathetic effect of dexmedetomidine.^13^ With in‐depth study of dexmedetomidine, in recent years, investigators have found that dexmedetomidine has a protective effect on organ injury.^14^, ^15^ Furthermore, both animal‐based and cell‐based experiments have confirmed that α_2_‐adrenoceptor agonists have an inhibitory effect on inflammatory responses^16^, ^17^, ^18^ both due to infectious and non‐infectious causes. Additionally, dexmedetomidine has been shown to reduce ischemia‐reperfusion injury in the heart^19^ and the brain,^20^ with main mechanism of reducing the inflammatory response mediated by oxygen free radicals and inflammatory factors.

At present, although dexmedetomidine has been commonly used as sedation therapy in ICU patients requiring MV, whether its efficacy and safety profile is superior to that of other drugs is still controversial. Some studies have shown that compared with midazolam or propofol, dexmedetomidine significantly shortens mechanical ventilation time and improves pain transmission in ICU patients.^21^, ^22^, ^23^, ^24^ On the contrary, other studies have revealed that the use of dexmedetomidine does not reduce mortality and ventilator‐free days in patients requiring MV.^25^, ^26^, ^27^ Based on the above controversies, the purpose of this study is to investigate the efficacy and safety of dexmedetomidine in patients undergoing MV by reviewing the results of randomized controlled trials (RCT).

## 2 MATERIALS AND METHODS

### 2.1 Retrieval strategy

PubMed (https://www.ncbi.nlm.nih.gov/pubmed/), EMbase (https://www.embase.com/), Web of Science (http://webofknowledge.com/), the Cochrane Library (https://www.cochranelibrary.com/), Medline (http://search.ebscohost.com/), OVID (http://ovidsp.dc2.ovid.com/) and (ClinicalTrials.gov https://clinicaltrials.gov/) were searched online to collect RCTs evaluating the efficacy and safety of dexmedetomidine in patients requiring MV. Retrieval was limited to the time‐frame between the establishment of the database to June 2019. In addition, the references included in the study were studied to supplement and obtain relevant articles The key words used in the retrieval process are as follows: dexmedetomidine, MPV‐1440, MPV 1440, Precedex, Dexmedetomidine Hydrochloride, Hydrochloride, Dexmedetomidine; Midazolam, Midazolam Maleate, Maleate, Midazolam, Dormicum, Versed, Midazolam Hydrochloride, Hydrochloride, Midazolam, Ro 21‐3981, Ro 21 3981, Ro 213 981; Propofol, 2,6‐Diisopropylphenol, Lorazepam, Ativan, Orfidal Wyeth, Wyeth, Orfidal, Témesta, Tolid, Donix, Duralozam, Durazolam, Idalprem, Laubeel, Lorazep Von Ct, Von Ct, Lorazep, Lorazepam Medical, Medical, Lorazepam, Lorazepam‐Neuraxpharm; mechanical ventilation; RCT.

### 2.2 Inclusion criteria

Following inclusion criteria were used; (a) participants aged ≥18 years; (b) participant requiring MV treatment, including invasive and non‐invasive MV; (c) RCT as the type of study; (d) the included articles provided enough information for subsequent summary analysis.

### 2.3 Exclusion criteria

Following exclusion criteria were used: (a) cross sectional studies, prospective cohort studies or retrospective cohort studies; (b) literature without relevant outcome indicators; (c) articles with original data unavailable or incomplete; (d) articles with data that cannot be summarized and analyzed; (e) articles on animal experiments; (f) republished literature.

### 2.4 Literature screening and data extraction

Two investigators separately screened the literature, extracted the relevant data and then cross‐checked the extracted data. In the event of disagreement between the two researchers, disagreements were resolved via discussion or negotiation with a third party. During literature screening, the title of the articles were read first. After excluding apparent irrelevant literature, the abstracts of remaining literature were read, followed by the full texts to determine if the articles met the inclusion criteria. If necessary, authors of the original research were contacted by email or telephone to obtain undetermined information considered of importance to the study. The extracted information is as follows: the name of the first author or trial, the number of years published, research design, the type of mechanical ventilation, age, sex, interventions and the final outcome to be used for analysis.

### 2.5 Quality evaluation of included literature

In this study, we used the Cochran risk bias assessment tool^28^ to evaluate the quality of the included literature.

### 2.6 Statistical analysis

In this study, Stata 11.0 software was used for statistical analysis. Risk ratio (RR) and 95% confidence interval (CI) were used as the effect indices for categorical variables. For continuous variables presenting as median and interquartile range (IQR)/median and standard deviation (SD), relevant data could not be extracted for subsequent meta‐analysis, since representing median as mean for comprehensive analysis can significantly increase the unreliability of the final result. When the median and IQR were converted to mean and SD according to previous results,^29^ the final result was apparently different from the included studies. Therefore, the results presenting as median and IQR/SD were analyzed descriptively. In this analysis, the random effect model was used to analyze the results comprehensively. The heterogeneity between the included studies was assessed using *I* ^2^ and *P*‐values. *P* > .1 or *I* ^2^ <50% suggested no significant heterogeneity between the included studies. In addition, sensitivity analysis was further done to explore the impact of individual studies on the final results and the Begg's test was performed to assess publication bias.

## 3 RESULTS

### 3.1 Flow chart of document retrieval process

**FIGURE 1:**
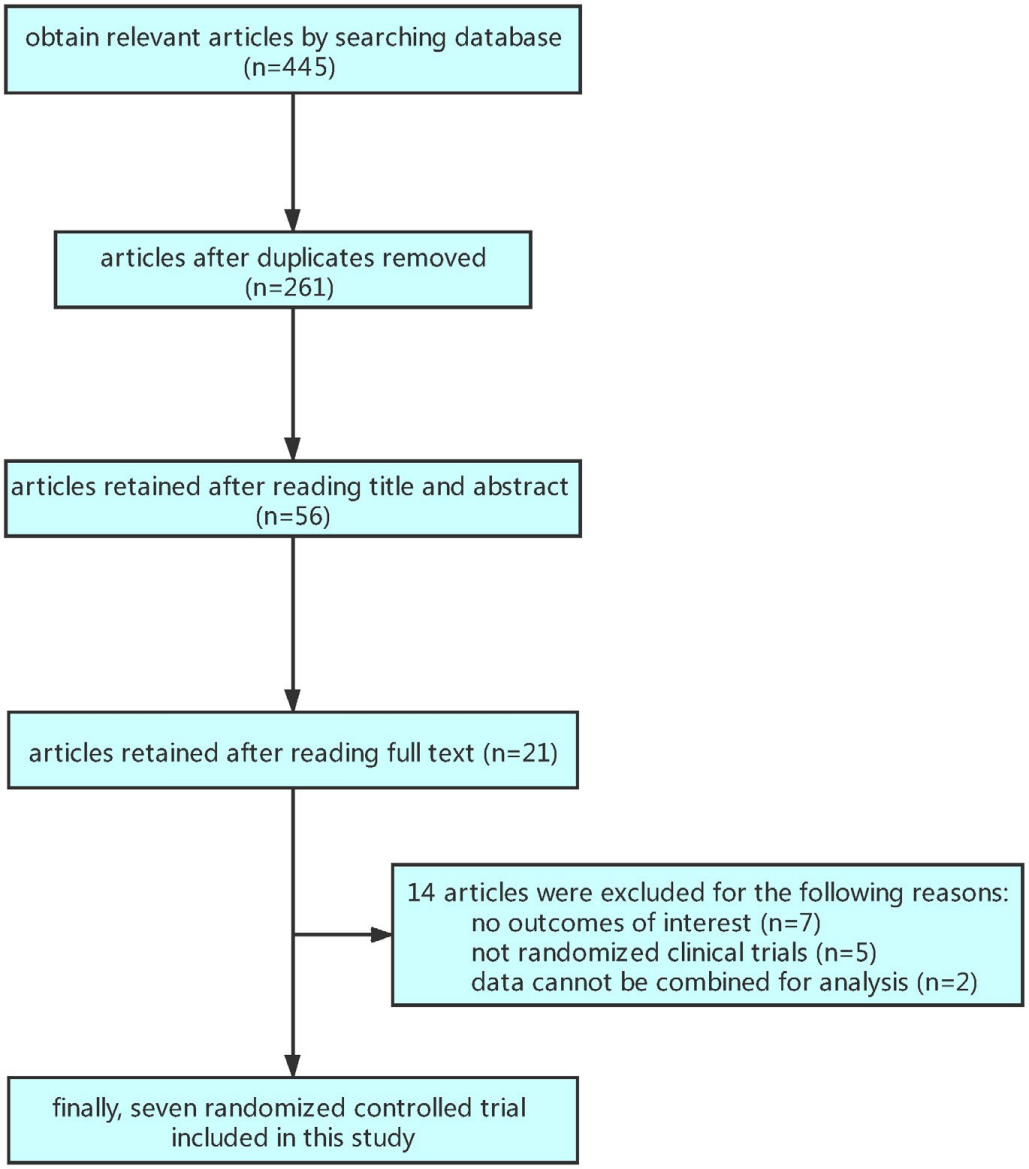
Flow chart of document retrieval process

First of all, we retrieved the relevant articles by key words, and then removed irrelevant articles upon reading the titles and the abstracts. Finally, the full texts were read to determine their suitability for inclusion in this study. The flow chart of literature retrieval is shown in Figure 1.

### 3.2 Basic characteristics of the included literature

The characteristics of the included articles are shown in Table 1. Seven RCTs^21^, ^22^, ^23^, ^24^, ^25^, ^26^, ^27^ were finally included in this summary analysis, with 986 participants in the dexmedetomidine group and 862 participants in the control group. Table 1 gives a detailed description of the basic characteristics of RTCs included, including publication years, age, gender, type of study and outcome of interest.

**TABLE 1:** Basic features of the included articles

Study	Year	Research design	MV type	Dexmedetomidine group			Control group			Outcome
				Age, y	Male, No. (%)	Interventions	Age, y	Male, No. (%)	Interventions	
Jakob SM	2012	RCT	IMV	65 (55‐74)/65 (51‐75)	153 (61.4)/160 (63.7)	Dexmedetomidine	65 (55‐74)/65 (51‐74)	175 (69.7)/166 (67.2)	Midazolam/propofol	ICU length of stay; AE
Kawazoe Y	2017	RCT	IMV/NIMV	68 (14.9)	63 (63)	Dexmedetomidine	69 (13.6)	64 (63)	Midazolam/propofol	28‐day mortality; VFD; ICU length of stay; AE
MENDS study	2007	RCT	MV	60 (49‐65)	30 (58)	Dexmedetomidine	59 (45‐67)	23 (45)	Lorazepam	28‐day mortality; ICU length of stay; VFD; delirium or coma
Riker RR	2009	RCT	IMV	61.5 (14.8)	125 (51.2)	Dexmedetomidine	62.9 (16.8)	57 (46.7)	Midazolam	ICU length of stay; delirium; 30‐day mortality
Ruokonen E	2009	RCT	MV	64 (18‐83)	32 (78.4)	Dexmedetomidine	68 (18‐83)	38 (86.3)	Midazolam/propofol	ICU length of stay;
Huang Z	2012	RCT	IMV	67.4 ± 8.2	12 (41.37)	Dexmedetomidine	61.5 ± 7.3	14 (42.4)	Midazolam	ICU length of stay; ICU mortality;
Devlin JW	2014	RCT	NIMV	68 ± 6	11 (69)	Dexmedetomidine	62 ± 17	6 (35)	Unclear	ICU mortality; ICU length of stay; delirium

Abbreviations: AE, adverse events; ICU, intensive care unit; IMV, invasive mechanical ventilation; MV, mechanical ventilation; NIMV, noninvasive mechanical ventilation; VFD, ventilator‐free days.

### 3.3 Quality evaluation of included studies

**FIGURE 2:**
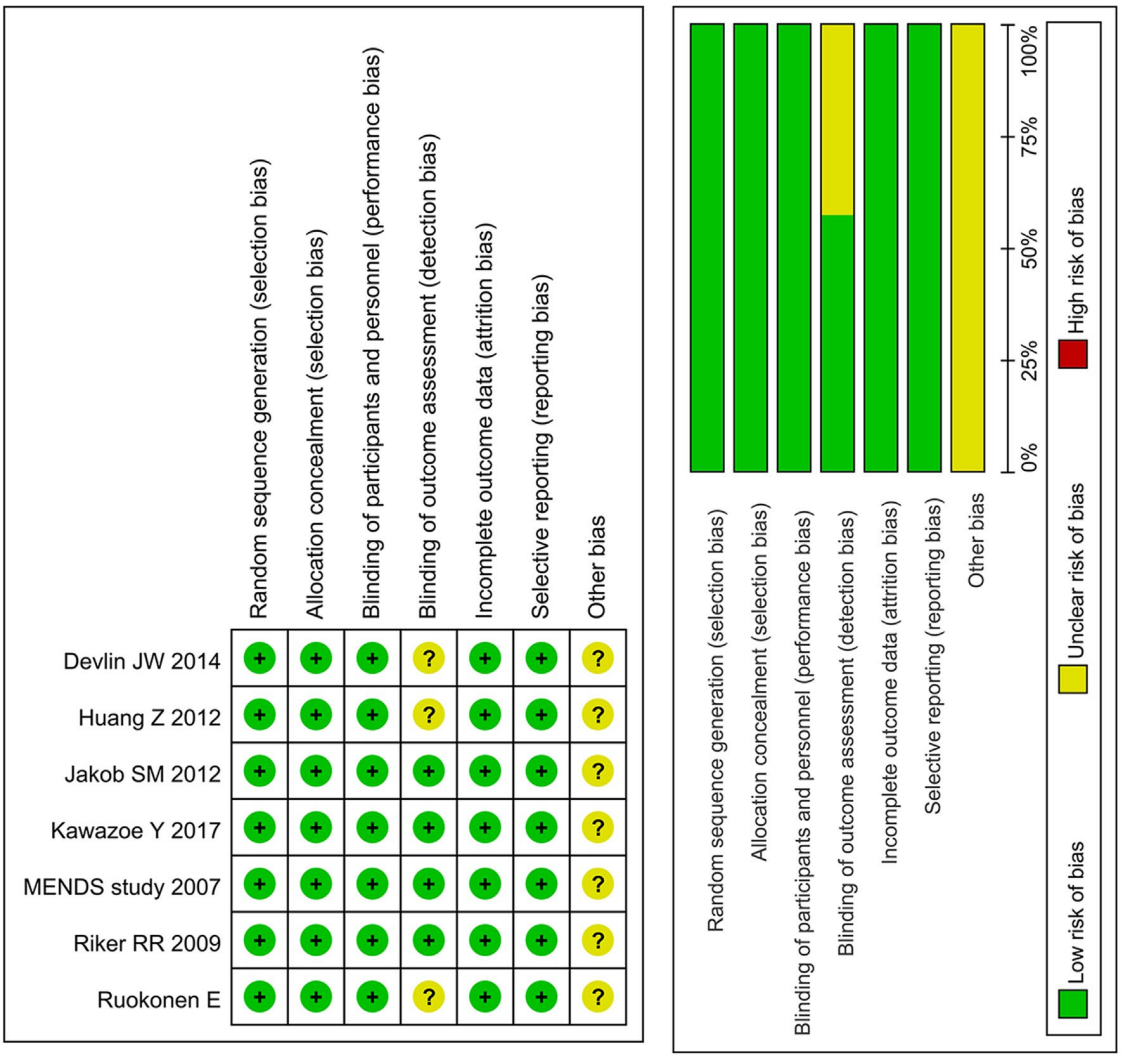
Quality evaluation of included studies

We used the Cochran risk bias assessment tool to evaluate the quality of the included literature, and the detailed evaluation of each study is shown in Figure 2.

### 3.4 Mortality at 30 days

**FIGURE 3:**
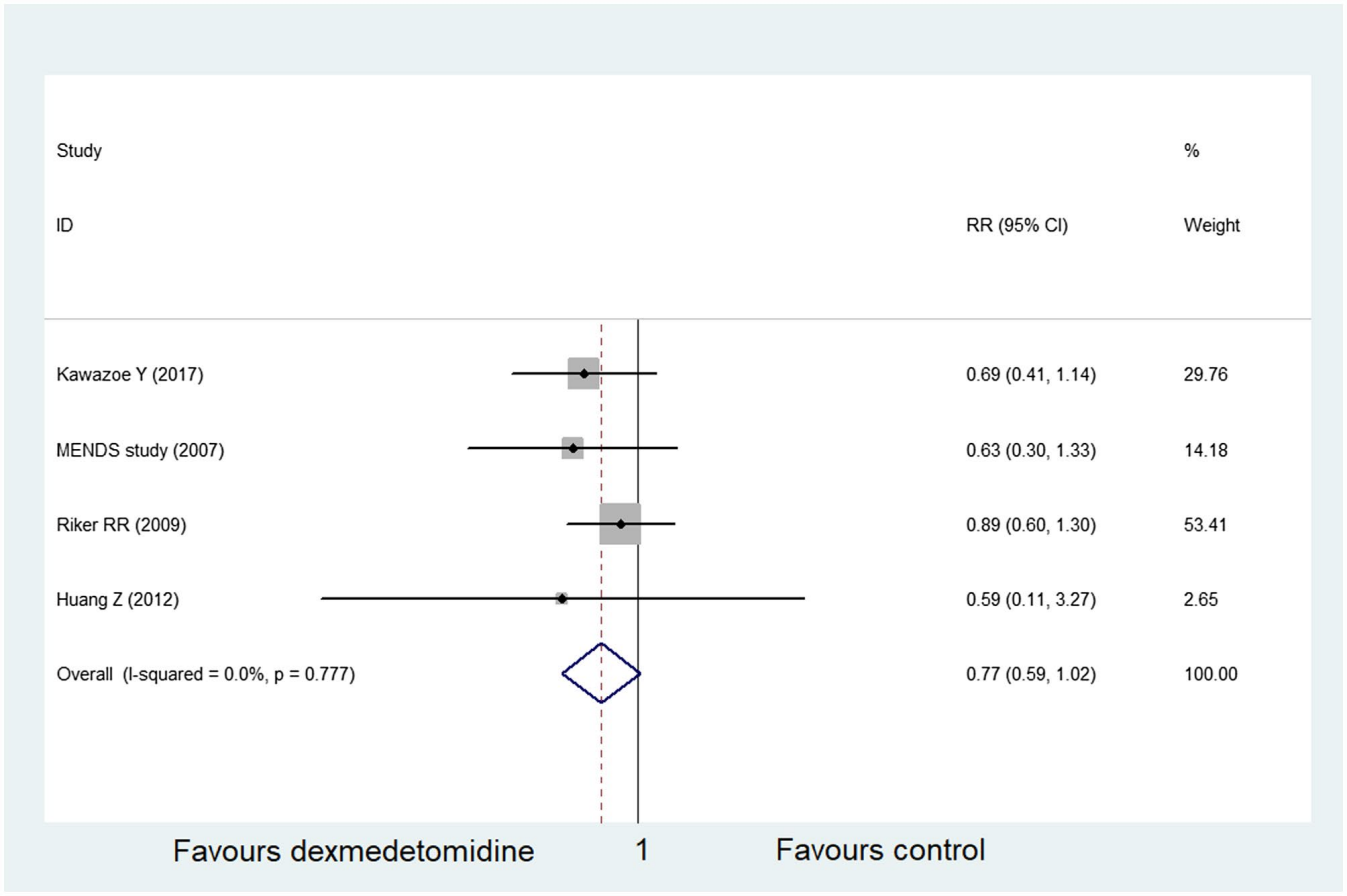
Comparison of mortality at 30 days between dexmedetomidine and the control group (forest plot)

**FIGURE 4:**
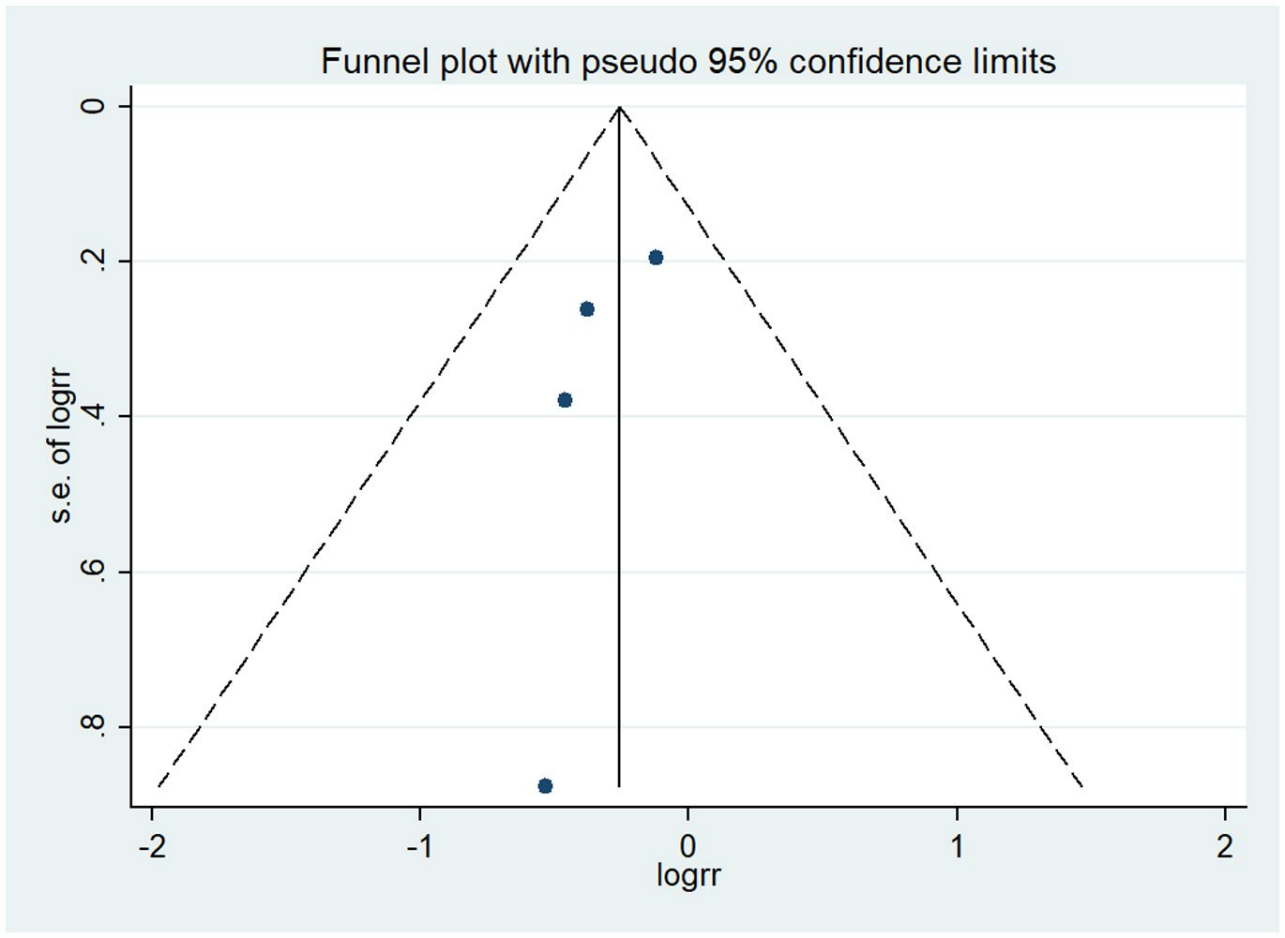
Comparison of mortality at 30 days between dexmedetomidine and the control group (funnel plot)

**FIGURE 5:**
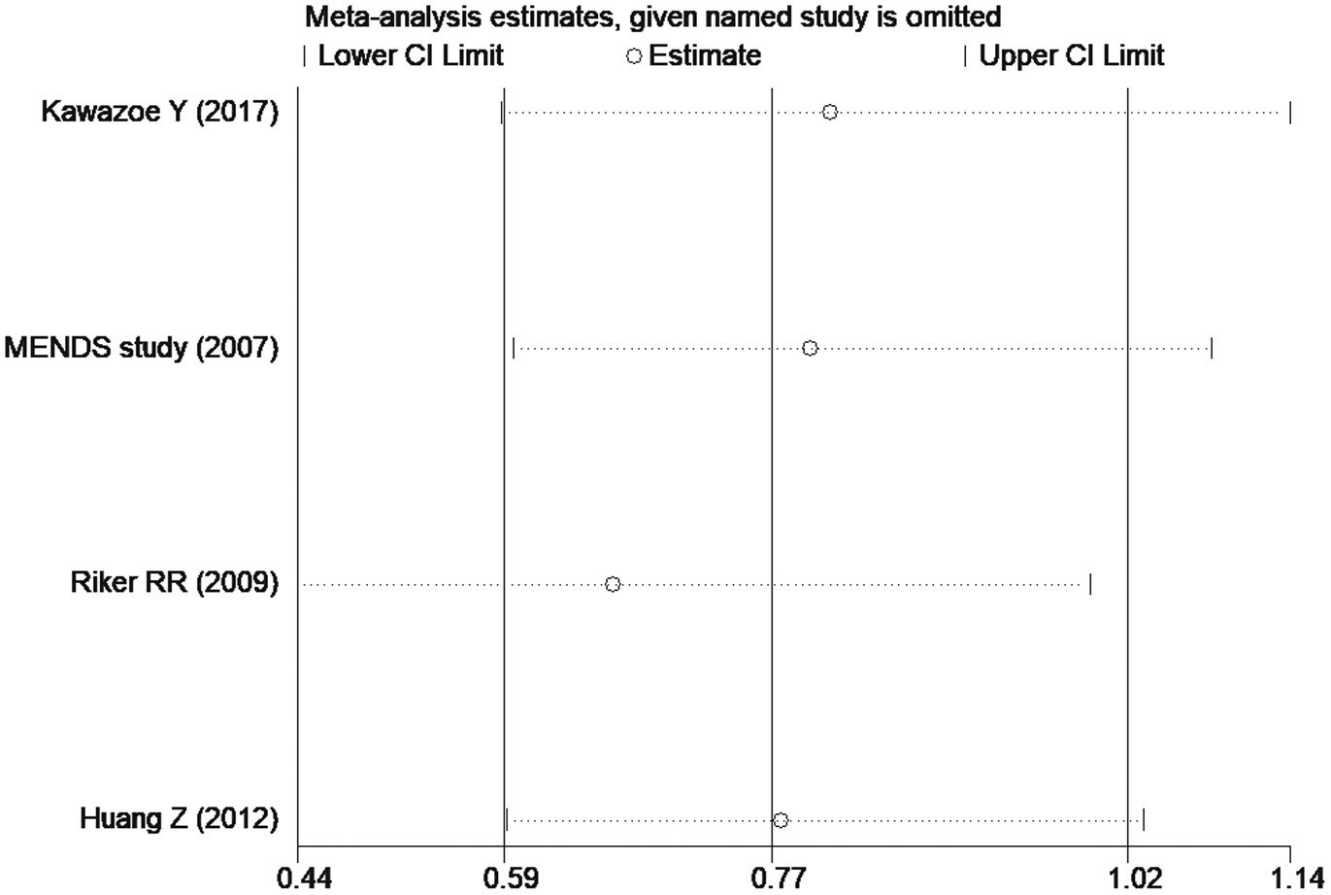
Comparison of mortality at 30 days between dexmedetomidine and the control group (sensitivity analysis)

**FIGURE 6:**
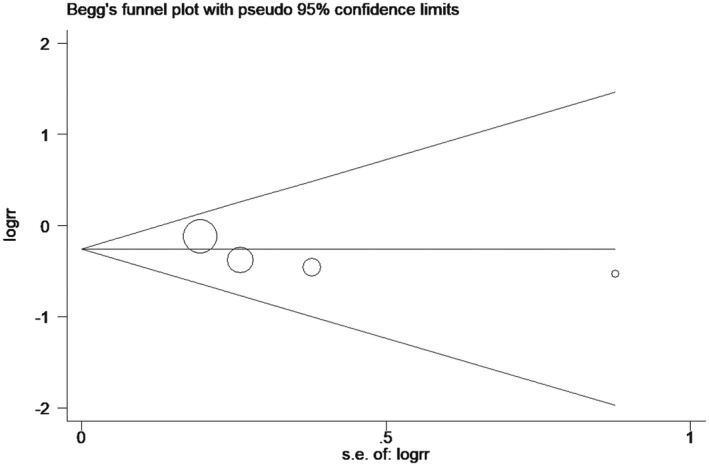
Comparison of mortality at 30 days between dexmedetomidine and the control group (publication bias)

In the final included literature, four studies^21^, ^23^, ^24^, ^26^ described 30‐day mortality, with a total of 429 participants in the dexmedetomidine group and 862 participants in the control group. The results of meta‐analysis displayed that dexmedetomidine was not superior to the control group in reducing 30‐day mortality (RR = 0.77, 95% CI: 0.59 to 1.02, *I* ^2^ = 0.0%) (Figure 3). Furthermore, the included studies showed no significant heterogeneity, as evident by the funnel plot and sensitivity analysis (Figures 4 and 5). In addition, no publication bias was found via Begg's test (Figure 6, *P* = .286).

### 3.5 Delirium

**FIGURE 7:**
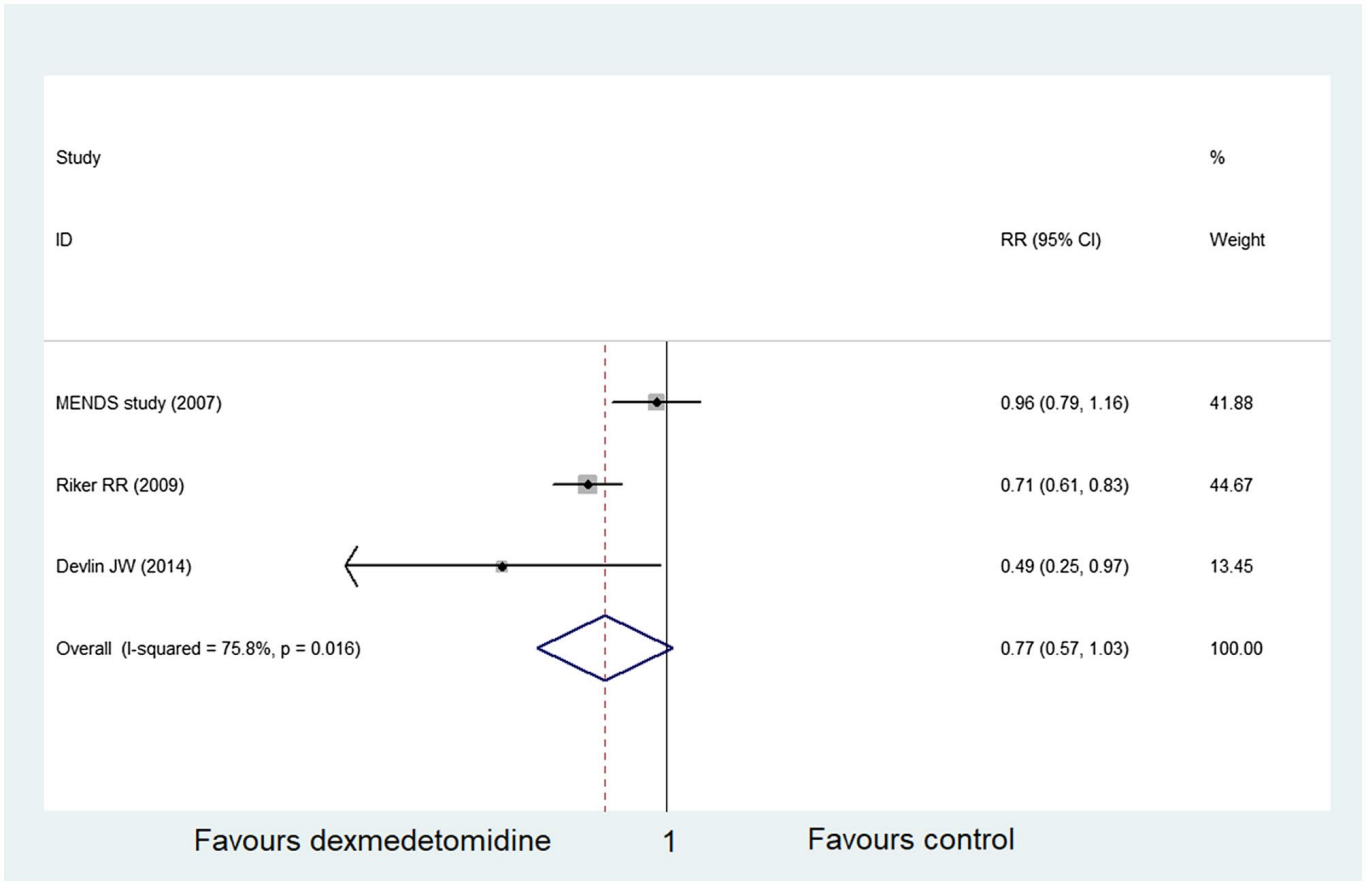
Comparison of delirium between dexmedetomidine and the control group (forest plot)

**FIGURE 8:**
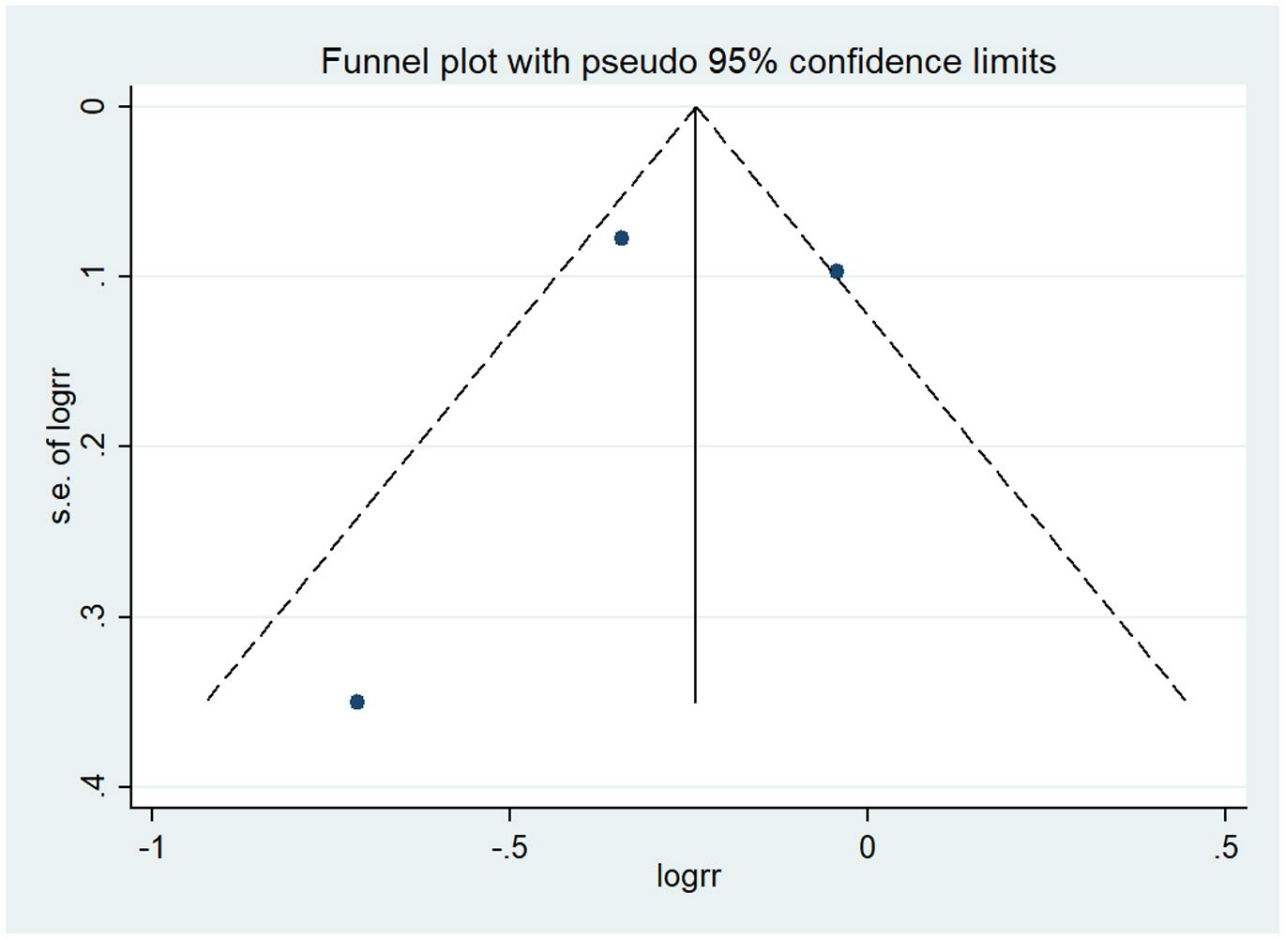
Comparison of delirium between dexmedetomidine and the control group (funnel plot)

**FIGURE 9:**
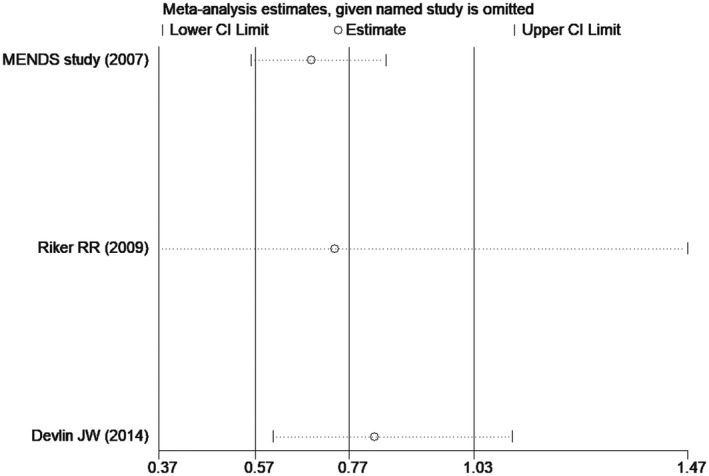
Comparison of delirium between dexmedetomidine and the control group (sensitivity analysis)

**FIGURE 10:**
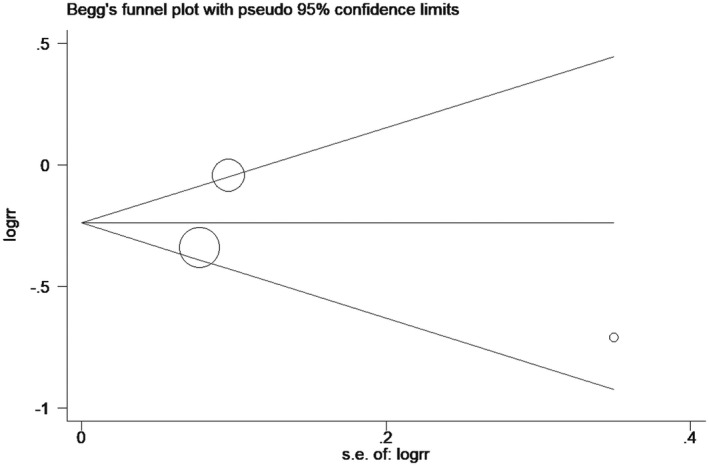
Comparison of delirium between dexmedetomidine and the control group (publication bias)

In the final included literature, three studies^23^, ^24^, ^25^ including 312 participants in the dexmedetomidine group and 190 participants in the control group, reported delirium. Summary analysis displayed no difference between dexmedetomidine and the control group in reducing the incidence of delirium (RR = 0.77, 95% CI: 0.57‐1.03, *I* ^2^ = 75.8%) (Figure 7). Similarly, the funnel plot and sensitivity analysis indicated that there was heterogeneity between the included studies, and that the heterogeneity mainly came from Riker RR study^24^ (Figures 8 and 9). Besides, the Begg's test revealed no publication bias among the included studies (Figure 10, *P* = .80).

### 3.6 Adverse events

**FIGURE 11:**
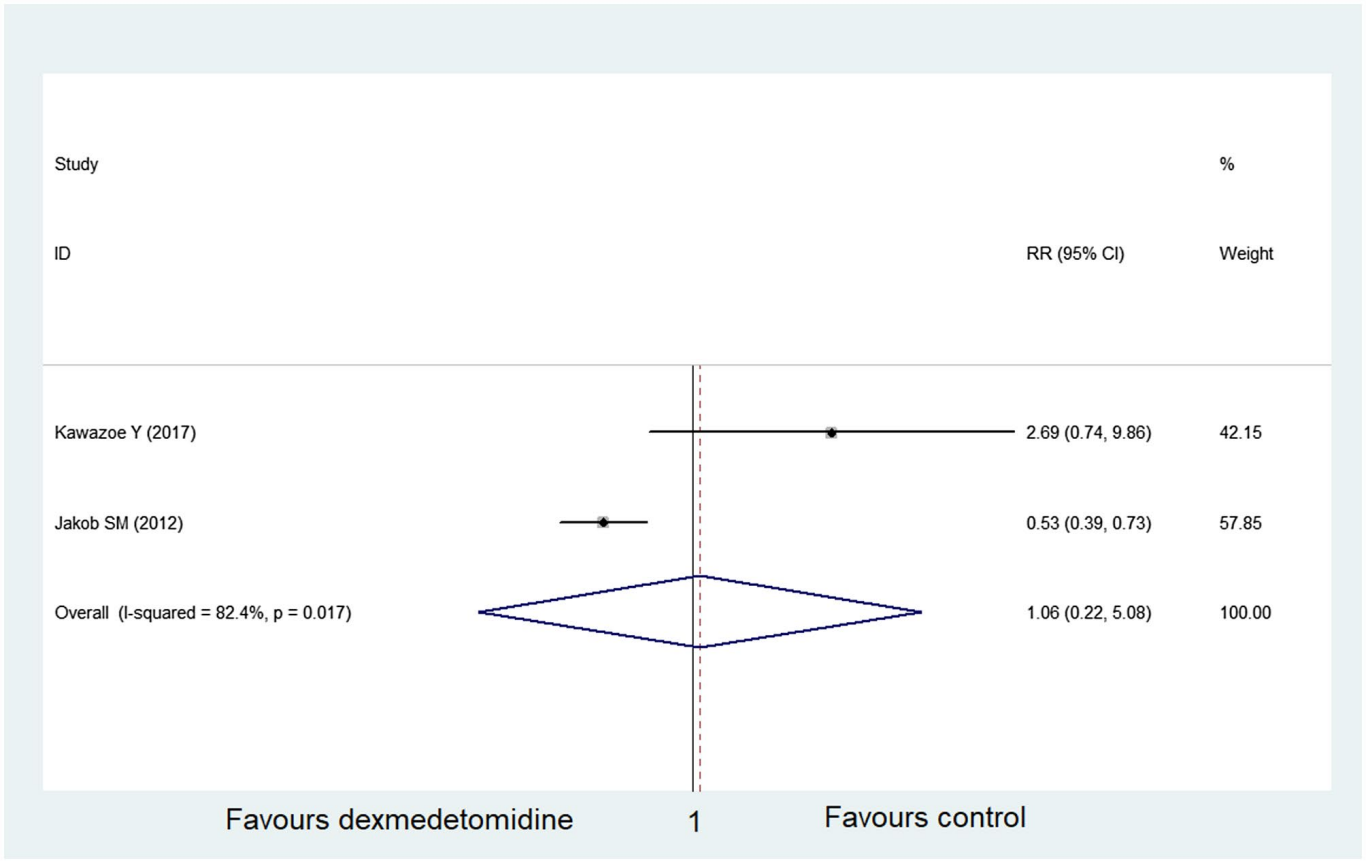
Comparison of adverse events between dexmedetomidine and the control group

In the final included literature, two studies,^22^, ^26^ including 600 participants in the dexmedetomidine group and 599 participants in the control group, described adverse events. Meta‐analysis showed that dexmedetomidine was not better than the control group in reducing the adverse events (RR = 1.06, 95% CI: 0.22‐5.08, *I* ^2^ = 82.4%) (Figure 11). No sensitivity analysis was performed, or bias tests published, due to the small number of studies involved.

### 3.7 ICU length of stay

In the final included literature, seven studies^21^, ^22^, ^23^, ^24^, ^25^, ^26^, ^27^ including 986 participants in the dexmedetomidine group and 862 participants in the control group, reported ICU length of stay. Additionally, in terms of ICU length of stay, all results (seven RCTs) were presented as median and IQR or median and SD. Therefore, for ICU length of stay, a descriptive analysis of the results was performed. Generally, for 99.65% (953/986) of patients, dexmedetomidine was not better than the control group in reducing ICU length of stay.

## 4 DISCUSSION

In recent years, although a variety of new drugs and combination treatments have been implemented to manage mechanically ventilated patients in ICU,^30^, ^31^, ^32^ decreasing the mortality and improving the short‐term and long‐term prognosis of mechanically ventilated patients still remains a great challenge, and therefore, an important problem needing urgent solution. Sedation therapy is an important treatment strategy to improve the prognosis of ICU patients requiring MV.^33^, ^34^ Our meta‐analysis showed that dexmedetomidine did not notably reduce 30‐day mortality, delirium, adverse events, and ICU length of stay in mechanically ventilated patients compared with the control group. Subsequent sensitivity analysis showed that there was certain heterogeneity in the included studies, however, no publication bias was found by Begg's test.

Most sedatives inevitably have certain cardiovascular side effects. Dexmedetomidine via its effect on the α 2A receptor distributed in the heart could lead to tachycardia and hypotension.^35^, ^36^ Previous studies have shown that dexmedetomidine increases the risk of bradycardia and hypotension in mechanically ventilated patients, and that the risk is approximately twice as high as that from midazolam or propofol,^22^ with the underlying cause related to the use of load and higher maintenance dose of dexmedetomidine. In this study, our results did not show dexmedetomidine leading to increase in adverse events of mechanically ventilated patients compared with the control. However, the results of our meta‐analysis still need to be viewed with caution, since the incidence of adverse events was not reported in detail in several studies, or alternatively some studies did not report information that could be extracted for statistical analysis. Therefore, the results of two RCTs were eventually used to evaluate the role of dexmedetomidine in increasing the incidence of adverse events in ICU patients requiring MV. In this process of statistical analysis, there may be selection bias.

Delirium^37^, ^38^ is a neurobehavioral syndrome with transient systemic disorder, induced by drugs, anxiety, hypoxia, metabolic abnormality, and inflammatory response. Currently, whether dexmedetomidine is superior to midazolam, propofol, or lorazepam in reducing the incidence of delirium in mechanically ventilated patients is still controversial. The results of the MENDS study^23^ indicated that in mechanically ventilated patients who received targeted sedation, the patients treated with dexmedetomidine experienced delirium for significantly longer time than the patients treated with lorazepam. Furthermore, the sedation state was better in patients treated with dexmedetomidine than lorazepam. Similarly, the conclusions of Riker RR^24^ also demonstrated that dexmedetomidine could decrease the rate of delirium in patients requiring MV compared with the control group. Conversely, another study^25^ advocated that dexmedetomidine was not superior to the control group in reducing the incidence of delirium in patients requiring MV. The results of our comprehensive analysis presented no difference between dexmedetomidine and the control in reducing the incidence of delirium. Similarly, this conclusion still needs to be viewed with caution, since invasive mechanical ventilation (IMV) or noninvasive mechanical ventilation (NIMV) may be a critical reason for inconsistent conclusions. In MENDS study,^23^ the type of MV (IMV or NIMV) was not reported in detail. Therefore, we were not able to conduct subgroup analysis based on the type of MV in our study. Hence, it is essential to pay close attention to the safety of dexmedetomidine in the sedative treatment of patients with mechanical ventilation.

The biological basis of dexmedetomidine in reducing mortality mainly comes from experimental evidence. That is, dexmedetomidine has a protective effect on brain tissue, myocardial and kidney injury,^39^, ^40^, ^41^ as well as the decrease of inflammatory factors and mortality^42^, ^43^ in animal models. Similar to previous results, our comprehensive analysis found that dexmedetomidine did not reduce the mortality of patients with mechanical ventilation compared with the control group (RR = 0.77, 95% CI: 0.59 to 1.02), suggesting that the experimental results may not be applied to human experiments. Our findings indicate that dexmedetomidine did not reduce ICU length of stay compared with the control group. Only one study showed that dexmedetomidine could reduce the length of stay in ICU, which can be explained as follows: First, the sample size of Huang Z's study is small and only 62 participants were included. The small size means that the study lacked sufficient statistical power. Second, population differences may be one of the reasons for the differences in results. For example, the study population of Huang Z's research is Chinese people, while the study population of other research is non‐Chinese people.

It should be emphasized that this study does have limitations, and they are as follows. First, for classified variables, we could accurately extract relevant data for subsequent meta‐analysis. However, for continuous variables, most of the results were expressed as median and IQR or median and SD, and therefore, could not be used for subsequent comprehensive analysis. In this case, only descriptive analysis could be conducted for such data. Second, it is generally known that the type of MV is closely related to final outcome. However, some included studies did not describe in detail whether patients used invasive or non‐invasive MV. Therefore, subgroup analysis could not be performed according to ventilator type. Third, we could not obtain the original data from the included studies for individual meta‐analysis. Fourth, although all the included studies were RCTs, there were still heterogeneity in the final results, suggesting that the conclusions made in this study still need to be viewed cautiously in clinical practice. Final, it should be emphasized that early published studies suggested that dexmedetomidine and control group have no significant difference in reducing delirium. However, recently published studies have shown that dexmedetomidine significantly reduces the incidence of delirium compared to control. Therefore, it is possible that with the increase in related studies, the results of comprehensive analysis might support that dexmedetomidine significantly reduces the incidence of delirium compared to control.

## 5 CONCLUSIONS

Our meta‐analysis indicated that compared with the control group, the dexmedetomidine group showed no statistically significant difference between mortality and prognosis in ICU patients requiring MV. However, in view of several limitations of this study, this conclusion still needs to be carefully adopted in clinical practice, and more RCTs are needed to validate this conclusion.

## 6 DATA SHARING AND DATA ACCESSIBILITY

All data generated and analyzed in the study are available from the corresponding author upon reasonable request.

## DISCLOSURES

The authors have no conflict of interest to declare.

## AUTHOR CONTRIBUTIONS

All authors participated in the whole process of this study and approved the final version.
